# Inter‐individual variation in SpO_2_ during endurance exercise in hypoxia does not correlate with endocrine and angiogenic growth factor responses

**DOI:** 10.14814/phy2.70221

**Published:** 2025-02-09

**Authors:** Hisashi Mori, Hyejung Hwang, Kazushige Goto

**Affiliations:** ^1^ School of Human Science and Environment University of Hyogo Himeji Hyogo Japan; ^2^ Faculty of Sport and Health Science Ritsumeikan University Kusatsu Shiga Japan; ^3^ Physical Activity and Performance Institute Konkuk University Seoul Republic of Korea

**Keywords:** cortisol, endurance training, growth hormone, oxygenation, variability

## Abstract

The present study determined the relationship between inter‐individual variation in arterial O_2_ saturation (SpO_2_) and exercise‐induced endocrine and angiogenic growth factor responses under hypoxia. Sixteen healthy men completed two trials on separate days: 60 min of cycling at 65% of maximal oxygen uptake (VO_2_max) followed by a 60‐min rest period, under either normoxia (FiO_2_ = 20.9%, NOR) or hypoxia (FiO_2_ = 14.5%, HYP). Serum growth hormone (GH), cortisol, and vascular endothelial growth factor (VEGF) concentrations were determined before, immediately after, and at 60 min after exercise. SpO_2_ and heart rate were continuously measured during exercise. In the HYP trial, the average SpO_2_ during exercise varied by >10% among all participants (77.5%–88.2%). However, the ΔSpO_2_ (Δ = HYP–NOR) did not correlate significantly with exercise‐induced changes in serum ΔGH (*r* = 0.205, *p* = 0.446), Δcortisol (*r* = 0.059, *p* = 0.828), and ΔVEGF (*r* = −0.004, *p* = 0.989). Moreover, no significant correlations were observed between the absolute SpO_2_ value and exercise‐induced responses in these blood variables in the HYP trial. Inter‐individual variation in SpO_2_ did not modify exercise‐induced endocrine (GH, cortisol) or angiogenic growth factor (VEGF) responses to endurance exercise in hypoxia.

## 1 INTRODUCTION

Exercise training in hypoxia (Living‐Low Training‐High) has been suggested as a potent procedure for promoting health (Morishima et al., 2014; Nishiwaki et al., 2011) and improving athletic performance (Dufour et al., 2006; Millet et al., 2010). In a study by Morishima et al. (2014), 20 sedentary participants underwent 60 min of cycling under hypoxia (*n* = 9, fraction of inspiratory oxygen [FiO_2_] = 15.0%) or normoxia (*n* = 11, FiO_2_ = 20.9%) for 4 weeks (3 days per week). The results revealed that the participants in the hypoxic training group exhibited significantly greater improvement in glucose tolerance compared with those in the normoxic training group. In terms of athletic performance, Dufour et al. (2006) demonstrated that 6 weeks of endurance training under hypoxia (FiO_2_ = 14.5%) resulted in greater increases in maximal oxygen uptake (VO_2_max) and time to exhaustion at constant‐velocity running than the same training under normoxia.

The adaptations resulting from endurance training in hypoxia can be explained by multiple factors. Decreases in O_2_ content in the blood circulation (i.e., systemic hypoxia) and muscle tissue (i.e., local hypoxia) are two key stimuli for initiating pathways associated with training adaptations (Vogt et al., 2001). In fact, lower muscle oxygenation level during exercise increases blood flow (increased shear stress for vascular endothelial cell) with vasodilatation mediated by nitric oxide production (Vedam et al., 2009), leading to growth factor secretion for angiogenesis (Wahl et al., 2013). Moreover, a lowered O_2_ content in blood circulation augments energy production via glycolysis (Bouissou et al., 1987; Katayama et al., 2010; Schmidt et al., 1995) and erythropoiesis (Chapman et al., 1998; Schmidt et al., 1991; Wahl et al., 2013), as well as growth hormone (GH) (Kon et al., 2015; Kurobe et al., 2015; Schmidt et al., 1995) and cortisol secretions (Kurobe et al., 2015). In particular, Wahl et al. (2013) reported that serum vascular endothelial growth factor (VEGF) concentration was significantly elevated after 90 min of cycling under hypoxia (FiO_2_ = 13.2%). Likewise, serum erythropoietin (EPO) concentration was elevated after exercise under hypoxia (FiO_2_ = 15.9% and 13.2%). Schmidt et al. (1995) also reported that blood lactate and serum GH elevations were augmented during 60 min of cycling under hypoxia (FiO_2_ = 12.1%) compared with levels in normoxia. Collectively, these results show that endurance exercise in hypoxia facilitates exercise‐induced hormonal and growth factor responses, and it also related to the severity of hypoxic levels.

However, there are large inter‐individual variations in endocrine responses to exercise in hypoxia (Chapman, 2013; Schmidt et al., 1995), and differences in arterial O_2_ saturation (SpO_2_) during exercise may be involved. Although SpO_2_ is a typical indicator of the severity of hypoxia in the body, large inter‐individual variations during endurance exercise occur even under the same FiO_2_ (Chapman, 2013; Costello et al., 2020). Thus, inter‐individual differences in SpO_2_ under identical hypoxic FiO_2_ may induce different endocrine and angiogenic growth factor responses.

Therefore, the purpose of the present study was to determine the relationship between inter‐individual variation in SpO_2_ and exercise‐induced endocrine or angiogenic growth factor responses following acute endurance exercise under hypoxia. We hypothesized that lower SpO_2_ during endurance exercise under hypoxia would be associated with augmented endocrine (i.e., GH, cortisol) and angiogenic growth factor (i.e., VEGF) responses.

## 2 METHODS

### 2.1 Participants

Sixteen healthy men (mean ± standard deviation [SD]: age 22 ± 2 years, height 174.6 ± 7.6 cm, body mass 67.8 ± 8.6 kg) participated in the present study. Participants had no history of exercise‐induced hypoxemia or iron‐deficiency anemia. All had lived their entire lives at sea level. The participants had different training backgrounds (e.g., sedentary, recreationally trained [baseball, soccer, long‐distance running], well‐trained [short and long sprint running]), ensuring large inter‐individual variation among the participants. The present study was approved by the Ethics Committee for Human Experiments at Ritsumeikan University, Japan (BKC‐IRB‐2016‐013). Written informed consent was obtained from each participant. All procedures were in accordance with the ethical standards of the institutional and/or national research committee and with the 1964 Helsinki Declaration and its later amendments or comparable ethical standards.

### 2.2 Experimental overview

Participants visited the laboratory four times during the experimental period. During the first and second visits, VO_2_max was assessed using a cycle ergometer (828E, Monark, Stockholm, Sweden), in randomized order, normoxia (FiO_2_ = 20.9%) or hypoxia (FiO_2_ = 14.5%). These tests were conducted on different days at least 1 week before the experimental trial.

On the third and fourth visits, two main trials were carried out in a single‐blind randomized cross‐over design. Trials were separated by 7~14 days. The experimental trials consisted of two different conditions: 60 min of exercise at 65% VO_2_max in normoxia (FiO_2_ = 20.9%, NOR) or 60 min of exercise at 65% VO_2_max in hypoxia (FiO_2_ = 14.5%, HYP). We chose exercise intensity at 65% VO_2_max (i.e., around anaerobic threshold) because this exercise intensity has been previously utilized for promoting health (Hayashi et al., 2018). On the two main trial days, the participants came to the laboratory at 8:00 a.m. following an overnight fast (at least 10 h after the previous meal). All exercise and measurements were completed in an environmental chamber with the room temperature and humidity set at 23°C and 50%, respectively. A whole‐room (14.8 m^2^) hypoxic chamber was used, and hypoxic conditions were created by insufflating nitrogen, as reported previously (Morishima et al., 2014; Kasai et al. 2019; Sumi et al., 2018). The room air in the chamber was circulated continuously. Oxygen and carbon dioxide concentrations within the chamber were monitored continuously. Participants were blinded to the fractional oxygen concentration in the chamber.

### 2.3 Measurements in preliminary trials

VO_2_max was measured on the cycle ergometer using a graded power test. The initial load was 60 W, and the load was increased progressively by 30 W every 2 min until the participant reached exhaustion. The test was terminated when the participants failed to maintain the prescribed pedaling frequency of 60 rpm or reached an oxygen consumption (VO_2_) plateau. Respiratory gases were collected and analyzed using an automatic gas analyzer (AE300S, Minato Medical Science Co., Ltd., Tokyo, Japan). The data collected were averaged every 30 s. Heart rate (HR) and arterial oxygen saturation (SpO_2_) were measured continuously during the test using a wireless HR monitor (RC3 GPS, Polar, Kempele, Finland) and a finger clip pulse oximeter (PULSOX‐Me300, TEIJIN, Tokyo, Japan), respectively. The VO_2_max in hypoxia (FiO_2_ = 14.5%) was measured on a separate day to determine the workload during an exercise trial in hypoxia.

### 2.4 Measurements in main trial days

**FIGURE 1:**
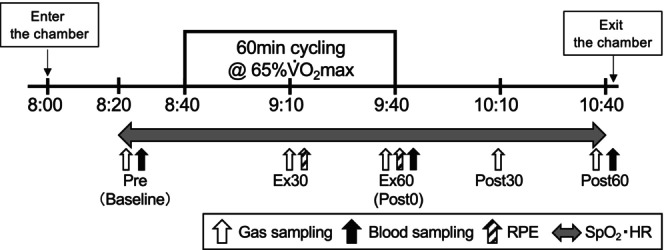
Experimental overview in main trial. Sampling point was indicated respiratory gas in the white arrow, blood in the black arrow, RPE in the black stripe arrow, and SpO2 and HR in the gray two‐way arrow.

Figure 1 shows the experimental overview on a main trial day. The participants visited the laboratory at 8:00 am and entered into the chamber under either normoxic or hypoxic conditions. After they rested at least 20 min in the chamber, respiratory gas, HR, and SpO_2_ were measured. Subsequently, a baseline blood sample was collected from the antecubital vein. The blood samples were collected at baseline, immediately (within 1 min) after exercise, and at 60 min after exercise. Blood drawings were conducted from a dominant arm. Respiratory gas was collected at baseline, at 25–30 min and 55–60 min during exercise, and at 55–60 min after exercise to determine VO_2_, carbon dioxide production (VCO_2_), minute ventilatory volume (VE), end‐tidal oxygen tension (PETO_2_), and end‐tidal carbon dioxide tension (PETCO_2_). All respiratory variables were collected using a breath‐by‐breath method and an automatic gas analysis system (AE300S, Minato Medical Science Co., Ltd.). The collected data were averaged over each 5‐min period. The respiratory exchange ratio (RER) was determined from the VO_2_ and VCO_2_ measurements. HR and SpO_2_ were continuously recorded at 1‐s intervals using a wireless HR monitor (RC3 GPS, Polar) and a finger clip pulse oximeter (PULSOX‐Me300, TEIJIN), respectively. Furthermore, respiratory strain (RPE‐R) and leg strain (RPE‐L) were measured every 10 min during the exercise using Borg's 6–20 scale for measuring perceived exertion (Borg, 1973; Borg et al., 2010).

### 2.5 Blood analysis

Blood samples were used to measure blood lactate, glucose, serum ferritin, iron, GH, cortisol, and VEGF concentrations. The serum ferritin was evaluated at baseline to confirm the participant's iron‐deficiency conditions. The serum samples were obtained by centrifugation (3000 rpm, 10 min, 4°C). The serum samples were stored at −60°C until analyses. Blood lactate and glucose concentrations were measured using an automatic lactate analyzer (Lactate Pro2, Arkray, Kyoto, Japan) and a glucose analyzer (Free Style, Nipro, Osaka, Japan), respectively. Serum iron, ferritin, GH, and cortisol concentrations were analyzed at the SRL Clinical Laboratory in Tokyo, Japan. Serum VEGF concentration was analyzed using an enzyme‐linked immunosorbent assay (ELISA) kit (DVE00, R&D Systems, Minneapolis, MN, USA). The intra‐assay coefficients of variation for each measurement were as follows: 1.6% for iron, 3.4% for ferritin, 2.9% for GH, 3.8% for cortisol, and 8.1% for VEGF.

### 2.6 Statistical analysis

Descriptive data are presented as the mean ± SD. The %VO_2_max and SpO_2_ values during 60 min of exercise were compared between the NOR and HYP trials using a paired *t*‐test, and time course changes in blood variables between the NOR and HYP trials were compared using two‐way (2 environments × 3 time points) repeated‐measures analysis of variance (ANOVA). When the ANOVA revealed a significant main effect or interaction, a paired *t*‐test was used to assess the differences. Comparisons of time course changes within each group were not made, as this was not the purpose of the present study.

The relationships between SpO_2_ values and blood variables were analyzed using Pearson's product moment correlation coefficients. To determine the difference of inter‐individual variation in SpO_2_ in the HYP trial on blood variables, all participants (*n* = 16) were assigned to two groups based on average SpO_2_ during the HYP trial. Thus, participants were assigned to a group with higher SpO_2_ (HIGH, *n* = 8; 83.8%–88.2%) or a group with lower SpO_2_ (Low, *n* = 8; 77.5%–83.6%). SpO_2_, respiratory variables, HR, and RPE during 60 min of exercise in hypoxia were compared between Low and High participants using an unpaired *t*‐test. Time course changes in blood variables in the HYP trial were initially analyzed using a two‐way (2 groups × 3 time points) repeated‐measures ANOVA. When the ANOVA revealed a significant interaction or main effect, an unpaired *t*‐test was used to assess the differences. No comparisons of time course changes were made, as this was not the purpose of the present study. The significance level was set at *p* < 0.05.

## 3 RESULTS

### 3.1 Comparisons of %VO_2_max, SpO_2_, and blood variables between NOR and HYP trials

Average values of SpO_2_ at baseline in NOR and HYP were 96.5 ± 1.4% (range: 93.9%–97.7%) and 87.5 ± 2.7% (range: 91.5%–82.4%), respectively, and SpO_2_ in HYP was significantly lower compared with NOR (*p* < 0.001). During 60 min of exercise, the average values of %VO_2_max (relative value for VO_2_max) were 67.1 ± 4.8% in the NOR trial and 67.3 ± 2.1% in the HYP trial; the difference was not significant (*p* = 0.862). As expected, average values of SpO_2_ during exercise were significantly lower in the HYP trial (83.2 ± 3.3%) than in the NOR trial (95.5 ± 0.9%, *p* < 0.01). Moreover, in the HYP trial, SpO_2_ values during exercise varied by >10% among all participants (77.5%–88.2%). The average HR during exercise was significantly higher in the NOR trial than in the HYP trial (150 ± 10 bpm and 145 ± 13 bpm, respectively; *p* = 0.048).

The analyses revealed no significant group effects or interactions (*p* = 0.38 and *p* = 0.11, respectively) for glucose, lactate, iron, GH, cortisol, or VEGF concentrations. There was no significant difference in pre‐exercise ferritin levels (*p* = 0.512; paired *t*‐test) between the NOR and HYP trials.

### 3.2 Relationship between SpO_2_ and blood variables in hypoxia

**FIGURE 2:**
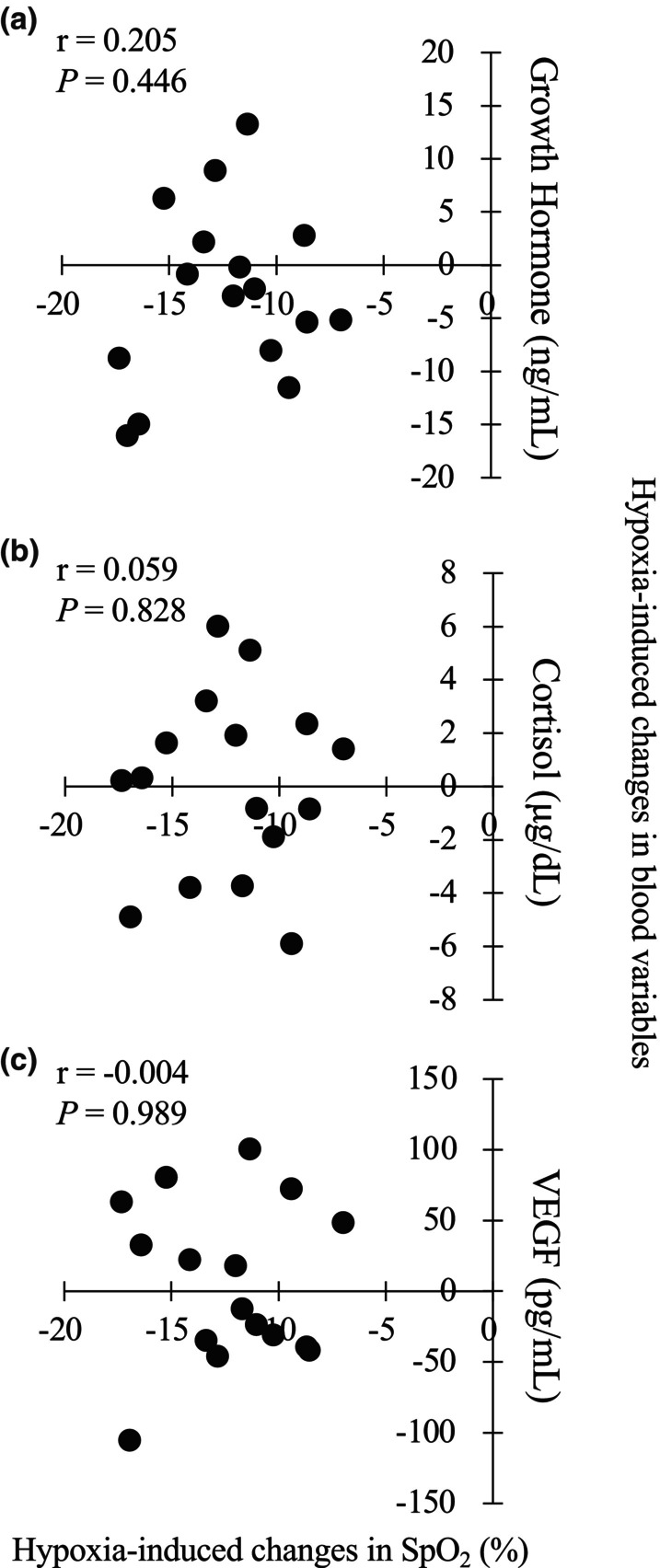
Relationships between hypoxia‐induced changes in SpO_2_ and endocrine responses (a) GH, (b) cortisol, (c) VEGF. Hypoxia‐induced changes were calculated as differences (HYP–NOR) in SpsO_2_ (*x*‐axis) or blood variables (*y*‐axis).

The ΔSpO_2_ at rest (SpO_2_ in HYP–SpO_2_ in NOR) was significantly correlated with the ΔSpO_2_ during exercise (*r* = 0.686, *p* = 0.03). Figure 2 shows the relationships between changes in SpO_2_ (ΔSpO_2_) and blood variables (i.e., GH, cortisol, and VEGF responses) immediately after exercise in the HYP trial. The ΔSpO_2_ was not significantly correlated with changes in serum GH (*r* = 0.205, *p =* 0.446), cortisol (*r* = 0.059, *p =* 0.828), or VEGF (*r* = −0.004, *p =* 0.989) concentrations. Likewise, ΔSpO_2_ was not significantly correlated with changes in blood glucose (*r* = 0.170, *p =* 0.530) or lactate (*r* = −0.147, *p =* 0.587) concentrations.

**FIGURE 3:**
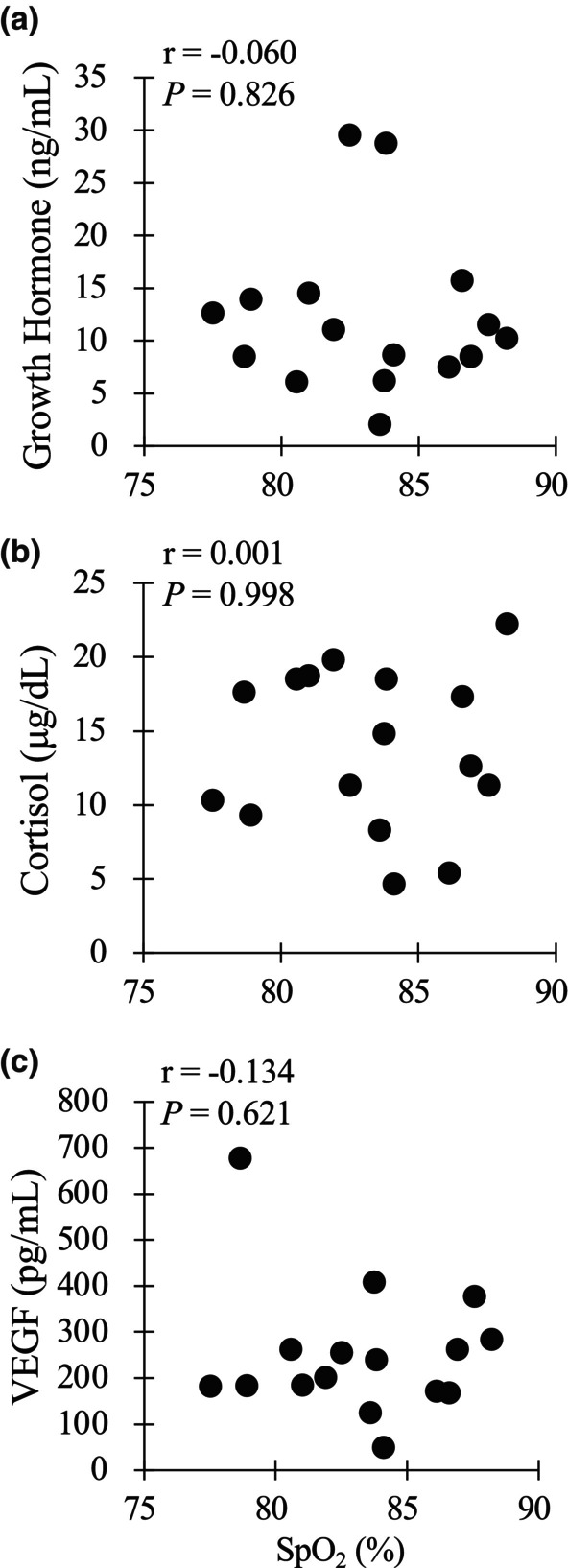
Relationship between absolute SpO_2_ values and absolute endocrine responses (a) GH, (b) cortisol, (c) VEGF immediately after exercise under hypoxia.

Figure 3 shows the relationship between SpO_2_ (absolute values) and blood variables (i.e., absolute concentrations of serum GH, cortisol, and VEGF) immediately after exercise in the HYP trial. The SpO_2_ was not significantly correlated with serum GH (*r* = −0.060, *p* = 0.826), cortisol (*r* = 0.001, *p =* 0.998), or VEGF concentrations (*r* = −0.134, *p =* 0.621). Similarly, SpO_2_ was not significantly correlated with blood glucose (*r* = −0.036, *p =* 0.894) or lactate (*r* = 0.087, *p =* 0.749) concentrations.

### 3.3 Comparison of respiratory and blood variables between Low and High participants

Table 1 shows a comparison of SpO_2_, respiratory variables, HR, and RPE during exercise (in the HYP trial) between Low and High participants. As expected, the mean SpO_2_ value was significantly lower (by 5.3%) in Low compared to High participants. The VO_2_ (*p* = 0.950), %VO_2_max (*p* = 0.804), VCO_2_ (*p* = 0.698), VE (*p* = 0.241), RER (*p* = 0.344), PETO_2_ (*p* = 0.108), HR (*p* = 0.290), RPE‐R (*p* = 0.835), and RPE‐L (*p* = 0.475) did not differ significantly between Low and High participants. By contrast, PETCO_2_ was significantly higher in Low participants (*p* = 0.043).

**TABLE 1:** Comparisons of SpO_2_, respiratory variables, HR, and RPE between LOW and HIGH participants during 60 min of exercise in hypoxia.

	Low (*n* = 8)	High (*n* = 8)	Significance
SpO_2_ (%)	80.6 ± 2.1	85.9 ± 1.8*	*p* < 0.01
VO_2_ (mL/min)	1745 ± 203	1751 ± 168	*p* = 0.950
%VO_2_ max (%)	67.2 ± 2.8	67.5 ± 1.3	*p* = 0.804
VCO_2_ (mL/min)	1612 ± 206	1647 ± 135	*p* = 0.698
VE (L/min)	56.0 ± 7.1	60.9 ± 8.8	*p* = 0.241
RER	0.92 ± 0.03	0.93 ± 0.05	*p* = 0.344
PETO_2_ (mmHg)	64.4 ± 2.9	64.3 ± 3.8	*p* = 0.108
PETCO_2_ (mmHg)	40.3 ± 1.1	37.7 ± 3.0*	*p* = 0.043
HR (bpm)	148 ± 16	141 ± 9	*p* = 0.290
RPE‐R	12.4 ± 1.9	12.2 ± 1.2	*p* = 0.835
RPE‐L	13.2 ± 1.7	13.7 ± 1.0	*p* = 0.475

*Note*: Respiratory variables were averaged at 30 min and 60 min during exercise. RPE‐R and RPE‐L were averaged at every 10 min during exercise. SpO_2_ and HR were averaged over the exercise period. Values are presented as the mean ± SD.

Abbreviations: %VO_2_max, percentage of maximal oxygen uptake; HR, heart rate; PETCO_2_, end‐tidal carbon dioxide tension; PETO_2_, end‐tidal oxygen tension; RER, respiratory exchange ratio; RPE‐L, rating of leg strain; RPE‐R, rating of respiratory strain; SpO_2_, arterial oxygen saturation; VCO_2_, carbon dioxide production; VE, ventilator volume; VO_2_, oxygen uptake.

* *p* < 0.05 versus Low.

Table 2 shows changes in blood variables in Low and High participants in the HYP trial. No significant interaction (2 groups × 3 time points) or main effect for group was found for changes in blood glucose (*p* = 0.620, 0.519), lactate (*p* = 0.938, 0.725), serum iron (*p* = 0.573, 0.132), GH (*p* = 0.753, 0.694), cortisol (*p* = 0.975, 0.585), or VEGF concentrations (*p* = 0.172, 0.955). There was no significant difference in pre‐exercise ferritin levels (*p* = 0.990; unpaired *t*‐test) between Low and High participants.

**TABLE 2:** Absolute changes in blood variables in Low and High participants under hypoxia.

		Pre	PostEx0	PostEx60	Interaction	Main effect (group)
Glucose (mg/dL)	Low	84 ± 6	80 ± 9	82 ± 6	*p* = 0.620	*p* = 0.519
	High	86 ± 3	84 ± 8	82 ± 7		
Lactate (mmol/L)	Low	1.3 ± 0.6	2.4 ± 0.9	1.6 ± 0.6	*p* = 0.938	*p* = 0.725
	High	1.3 ± 0.5	2.4 ± 0.8	1.5 ± 0.2		
Ferritin (ng/mL)	Low	61.0 ± 37.3				
	High	60.8 ± 36.1				
iron (μg/dL)	Low	136 ± 45	159 ± 48	154 ± 47	*p* = 0.573	*p* = 0.132
	High	99 ± 43	116 ± 56	116 ± 57		
GH (ng/mL)	Low	2.1 ± 3.5	12.3 ± 8.1	1.7 ± 1.2	*p* = 0.753	*p* = 0.694
	High	3.8 ± 4.8	12.1 ± 7.3	2.0 ± 1.1		
Cortisol (μg/dL)	Low	13.1 ± 1.7	14.2 ± 4.8	12.2 ± 3.7	*p* = 0.974	*p* = 0.585
	High	12.0 ± 2.6	13.3 ± 6.2	11.2 ± 4.8		
VEGF (pg/mL)	Low	227 ± 152	258 ± 175	176 ± 152	*p* = 0.172	*p* = 0.955
	High	204 ± 91	244 ± 117	201 ± 99		

*Note*: Values are presented as the mean ± SD. Low: *n* = 8, High: *n* = 8.

Abbreviations: GH, growth hormone; PostEx0, immediately after exercise (within 1 min); PostEx60, 60 min after exercise; Pre, Baseline; VEGF, vascular endothelial growth factor.

## 4 DISCUSSION

It was notable that SpO_2_ in the HYP trial varied by more than 10% among participants (from 77.5% to 88.2%) despite the identical FiO_2_ (14.5%) during the endurance exercise. However, the ΔSpO_2_ (SpO_2_ in HYP–SpO_2_ in NOR) was not significantly correlated with exercise‐induced changes in serum GH, cortisol, or VEGF concentration. Moreover, no significant correlation was observed between the absolute SpO_2_ value and exercise‐induced changes in these blood variables in the HYP trial. In addition, there were inter‐individual variations in exercise‐induced hormonal responses regardless of degree of hypoximia. Therefore, we also compared exercise‐induced serum GH, cortisol, and VEGF concentrations between Low (participants with lower SpO_2_) and High (participants with higher SpO_2_) participants, and found that these variables did not differ significantly between the two groups. These results suggest that large inter‐individual variation in SpO_2_ did not correlate endocrine (i.e., GH, cortisol) or angiogenic growth factor (i.e., VEGF) responses to endurance exercise in hypoxia.

Several previous studies have reported that endocrine (e.g., GH, cortisol, EPO) and angiogenic growth factor (i.e., VEGF) responses were augmented during exercise under hypoxia (Chapman et al., 1998; Ge et al., 2002; Kon et al., 2015; Kurobe et al., 2015; Schmidt et al., 1991, 1995; Wahl et al., 2013). Wahl et al. (2013) reported that the serum VEGF concentration was significantly elevated after 90 min of cycling exercise under hypoxia (FiO_2_ = 13.2%). In addition, Ge et al. (2002) investigated the relationship between hypoxia‐induced desaturation (ΔSaO_2_) and EPO responses (ΔEPO) and found that ΔSaO_2_ was significantly correlated with ΔEPO. In the present study, we hypothesized that oxygen desaturation in the blood under hypoxia (indicated by lower SpO_2_) would augment exercise‐induced endocrine responses, but the hypothesis was not supported.

We also compared exercise‐induced endocrine and angiogenic growth factor responses between participants with lower SpO_2_ (Low) and those with higher SpO_2_ (High) to clarify the impact of inter‐individual variation in SpO_2_ during endurance exercise in hypoxia. First, cardiorespiratory variables (e.g., VO_2_, %VO_2_max, VCO_2_, VE, RER, PETO_2_, PETCO_2_, and HR) were not significantly different between Low and High participants, except for PETCO_2_. These results indicate that the relative exercise intensity and cardiorespiratory strain during endurance exercise were matched between the two groups. Of particular note, RPE reflects central command activity, which promotes GH production (Franke et al., 2000), and this did not differ between the two groups. We then compared exercise‐induced GH, cortisol, and VEGF elevations between High and Low participants. No significant group effects or interactions were found, despite a 5.3% difference in average SpO_2_ between the two groups. Therefore, variation in SpO_2_ during endurance exercise in hypoxia did not affect endocrine or angiogenic growth factor responses.

Although SpO_2_ is widely used for monitoring the severity of hypoxia in the body, it does not sufficiently reflect oxygen content in arterial blood and muscle tissue oxygenation (Ge et al., 2002; Osawa et al., 2017). In fact, a previous study demonstrated an inconsistent relationship between SpO_2_ and muscle tissue oxygenation (Osawa et al., 2017). During endurance exercise in hypoxia, hypoxia inducible factor‐1 (HIF‐1) is a key regulator that elicits several metabolic cascades, such as angiogenic growth factor production, glycolysis, and erythropoiesis (Fandrey, 2004; Ohno et al., 2012). However, the HIF‐1 response is also regulated by cell oxygenation level (Stroka et al., 2001; Vogt et al., 2001). Therefore, in the present study, it is plausible that the blood oxygen desaturation level (expressed by ΔSpO_2_ or absolute SpO_2_) did not correlate muscle tissue oxygenation level during endurance exercise. Hence, further investigation to determine the relationship between muscle oxygenation and endocrine regulation is required.

A typical program of exercise training in hypoxia for improving exercise performance or health would include moderate (FiO_2_ between 14.5% and 16.4%) hypoxia (Dufour et al., 2006; Girard et al., 2017; Millet et al., 2010; Morishima et al., 2014). Therefore, the present study was conducted using a FiO_2_ of 14.5% based on these previous studies. On the other hand, other studies have reported that endocrine and angiogenic growth factor responses following exercise in hypoxia were dependent on the severity of hypoxia, and severe hypoxia (FiO_2_ < 13.5%) during exercise maximized these responses (Kon et al., 2015; Kurobe et al., 2015; Schmidt et al., 1995; Wahl et al., 2013). In the present study, the average SpO_2_ during exercise in the LOW group was 80.6%, which was higher than the values observed in previous studies (SpO_2_ 70%–75%) (Kon et al., 2015; Wahl et al., 2013). Therefore, the lack of differences in endocrine and angiogenic growth factor responses may be attributable to an insufficient decrease in SpO_2_ during exercise.

Several limitations exist when interpreting the present results. SpO_2_ is a typical indicator of severity of hypoxia. However, while SpO_2_ reflects the ratio of oxygenated to total hemoglobin in arterial blood, it does not reflect completely the true O_2_ demand or supply within the body. Thus, it would be necessary to evaluate O_2_ content in the blood (i.e., CaO_2_) in future study.

In conclusion, there were large inter‐individual variations in SpO_2_ during exercise under hypoxic conditions. However, inter‐individual variations in SpO_2_ did not correlate exercise‐induced endocrine (i.e., GH, cortisol) or angiogenic growth factor (i.e., VEGF) responses. These results suggest that SpO_2_ during exercise under hypoxia is not related to the magnitude of endocrine and angiogenic growth factor responses.

## AUTHOR CONTRIBUTIONS

The study was designed by Hisashi Mori, and Kazushige Goto. Material preparation, data collection and analysis were performed by Hisashi Mori, Hyejung Hwang. The first draft of the manuscript was written by Hisashi Mori, and Kazushige Goto. All authors read and approved the final manuscript.

## FUNDING INFORMATION

The present study was funded by Grant‐in‐Aid for Scientific Research from the Japan Society for the Promotion of Science (No. 16 J07109).

## CONFLICT OF INTEREST STATEMENT

The authors declare no conflicts of interest.

## ETHICS STATEMENT

The data are not publicly available due to ethical restrictions.

## Data Availability

The data presented in this study are available on request from the corresponding author.
